# Chronic pain experience and health inequities during the COVID-19 pandemic in Canada: qualitative findings from the chronic pain & COVID-19 pan-Canadian study

**DOI:** 10.1186/s12939-021-01496-1

**Published:** 2021-06-23

**Authors:** Lise Dassieu, M. Gabrielle Pagé, Anaïs Lacasse, Maude Laflamme, Vickie Perron, Audrée Janelle-Montcalm, Maria Hudspith, Gregg Moor, Kathryn Sutton, James M Thompson, Manon Choinière

**Affiliations:** 1 Research Center of the Centre Hospitalier de l’Université de Montréal (CRCHUM), Saint- Antoine Building, 850 Saint Denis St, H2X 0A9 Montreal, Quebec Canada; 2 Faculty of Medicine, Department of Anesthesiology and Pain Medicine, Université de Montréal, Pavillon Roger-Gaudry, succursale Centre-ville, C.P. 6128, H3C 3J7 Montreal, Québec Canada; 3 Faculty of Arts and Science, Department of Psychology, Pavillon Marie- Victorin, Université de Montréal, 90 avenue Vincent d’Indy, H2V 2S9 Montreal, Québec Canada; 4 Department of Health Sciences, Université du Québec en Abitibi-Témiscamingue (UQAT), Rouyn-Noranda, Quebec Canada; 5 Faculty of Arts and Sciences, Department of Sociology, Université de Montréal, H3C 3J7 Montreal, Québec Canada; 6 Pain BC Society, Vancouver, British Columbia Canada; 7 Department of Public Health Sciences, Queens University, Kingston, Ontario Canada; 8 Department of Family Medicine, Dalhousie University, Halifax, Nova Scotia Canada

**Keywords:** Chronic pain, COVID-19, Social inequities, Illness experience, Qualitative research, Canada

## Abstract

**Background:**

Chronic pain affects about 20 % of the Canadian population and can lead to physical, psychological and social vulnerabilities. However, this condition remains poorly recognized and undertreated. During 2020, as the COVID-19 pandemic disrupted daily living and health care systems, the situation of people with chronic pain has drawn little public attention.

**Methods:**

This qualitative study was part of a pan-Canadian mixed-methods project and aimed to understand the experiences and challenges of people living with chronic pain during the COVID-19 pandemic in Canada. Between May and August 2020, we conducted in-depth semi-structured interviews with 22 individuals living with chronic pain across the country. We used reflexive thematic analysis to interpret data.

**Results:**

Our findings underscored four dimensions of the chronic pain experience during the pandemic: (1) Reinforced vulnerability due to uncertainties regarding pain and its management; (2) Social network as a determinant of pain and psychological condition; (3) Increasing systemic inequities intermingling with the chronic pain experience; (4) More viable living conditions due to confinement measures. Though several participants reported improvements in their quality of life and reduced social pressure in the context of stay-at-home orders, participants from socio-economically deprived groups and minorities reported more challenges in accessing pain relief, health care services, and psychosocial support.

**Conclusions:**

The COVID-19 pandemic has revealed and intensified pre-existing disparities and challenges among people living with chronic pain in terms of material resources, psychosocial condition, social support, and access to care. In post-pandemic times, it will be essential to address flaws in health and welfare policies to foster equity and social inclusiveness of people with chronic pain.

## Background

During 2020, the COVID-19 pandemic disrupted everyday life in most countries in the world, leading to major physical, psychological, and socio-economic impacts in the general population [1–3]. In Canada, the provinces that were the hardest hit in terms of COVID-19 infection cases and deaths were Quebec (303,051 cases and 10,614 deaths as of March 22, 2021), Ontario (330,573 cases and 7,224 deaths as of March 22, 2021), and Alberta (142,390 cases and 1,968 deaths as of March 22, 2021), within a total of 938,719 cases and 22,716 deaths in the country [4]. As in many other countries, unprepared health care systems have been under pressure due to the sudden influx of patients presenting with COVID-19 and dealing with complications arising from this disease [5, 6]. The need to manage serious COVID-19 patients created challenges to provide usual levels of and access to healthcare services, including the treatment of chronic health conditions [7–9].

The pandemic has led to intensifying or making more visible, the pre-existing socio-economic and systemic inequities. Groups with lower socio-economic status, immigrants, and racialized persons were especially exposed to the virus as a result of precarious housing conditions and/or work in essential services [10–12]. They also faced additional economic burdens such as job loss or evictions [3, 10–15]. In addition, many people that did not previously belong to socioeconomically disadvantaged groups fell into poverty as a result of the unintended economic consequences of the pandemic [3].

Chronic pain, defined as persistent or recurrent pain for more than three months [16], affects approximately 20 % of the population in Canada [17], Europe [18], and the United States [19], and its prevalence is even higher in some other regions of the world such as Africa and Asia [20, 21]. Chronic pain can result from various sources such as accidents, surgeries, diseases, or unidentified causes [16] and can most likely lead to significant negative consequences on physical and mental health as well as social and professional life [18, 22]. However, this chronic condition remains largely undertreated and poorly recognized in public health policies [23–25]. In addition, social disparities regarding chronic pain have been observed in the literature: socially marginalized populations [26–28], women [29–31], and members of racial minorities [32–34] are more frequently affected and more likely to face stigma and challenges in accessing pain diagnosis and treatment.

The specific consequences of the COVID-19 pandemic on people living with chronic pain have not drawn much attention from policymakers, despite some pain clinicians and researchers sounding the alarm regarding the numerous vulnerabilities of these patients [35–38], and the need to ensure continuity of pain management during the pandemic [39–41]. A survey among Canadian pain clinics underscored major disruptions of services offered in these facilities during the COVID-19 first wave [42]. A few recent quantitative studies in Spain and North America [43–46] have identified some negative impacts of the pandemic on pain and its management, such as pain deterioration due to increased stress and anxiety [43–45] and changes in pain treatments [43, 46]. However, currently, no qualitative studies have examined the impacts of the pandemic from the perspectives of individuals living with chronic pain.

The objective of the present qualitative study was to provide an in-depth understanding of the daily experience of Canadians living with chronic pain during the COVID-19 pandemic, including their experience of pain and its management, psychological condition, and impact on social life. In order to understand the impacts of the pandemic from the subjective perspectives of participants, this study drew from the theoretical framework of sociology of the chronic illness experience [47, 48], which focuses on “people’s everyday lives living with and in spite of illness” and “the social organization of the sufferer’s world” [47]. When appropriate, the theoretical framework of social inequities in health and health care [49, 50] enabled us to situate participants’ individual experiences and narratives in the context of systemic dynamics and the social determinants of health. Indeed, it was important to avoid interpreting participants’ experiences exclusively with an individual-level lens. In addition to scientific knowledge advancements in the area of health inequities, we expect our findings to contribute to more equitable clinical and policy responses to the pandemic, in line with the diverse experiences and challenges of people living with chronic pain.

## Methods

### Study design

This study is the qualitative component of a convergent mixed-methods project examining the impact of the COVID-19 pandemic and related public health measures on Canadians living with chronic pain: the Chronic Pain & COVID-19 Pan-Canadian Study [43, 46]. The quantitative component included an online survey aimed at exploring changes in participants’ pain severity, pain treatment, and psychological well-being during the pandemic. To provide a more comprehensive insight into participants’ experiences and perspectives, this qualitative study was conducted using online in-depth interviews with participants in the quantitative survey. The Ethics Committee of the *Centre Hospitalier de l’Université de Montréal* approved the study.

### Recruitment and participants

Between April 15th and May 31st, 2020, an online questionnaire was disseminated among people living with chronic pain across Canada. A purposive sample of 22 persons was recruited among the respondents in this survey who consented to be contacted for an interview [1752 (66,3 %) of the 2644 participants who completed the survey consented to be contacted]. Qualitative interviews took place between May 12th and August 7th, 2020, –i.e. at the end of COVID-19 first wave and the beginning of service reopening in Canada. Throughout the recruitment process, 37 eligible participants were contacted through an invitation e-mail and 22 of them were effectively interviewed. Ten never responded to our emails and reminders, five declined or did not show up.

The study inclusion criteria were similar to those used in the quantitative component of the project [43, 46], namely (1) living with pain for longer than three months, (2) living in Canada, (3) being 18 years or older, (4) being fluent in English or French, and (5) being able to provide consent using an online form. To embrace a wide range of situations [51, 52], we diversified the sample according to age, province of residence, gender identity, ethnicity (including Indigenous Peoples of Canada identification), and living area (rural/urban). Rather than seeking representativeness of our larger quantitative sample, our sampling methodology was aimed at maximizing socio-demographic diversity to examine experiences of participants that were insufficiently represented in our statistical analysis (e.g. racial minorities).

Sociodemographic and pain-related characteristics of participants appear in Table 1. None of the participants had a COVID-19 diagnosis confirmed with a test. However, four participants experienced COVID-19-like symptoms prior to the interview. Two had a test returning negative results; one could not access testing but got a COVID-19 diagnosis after clinical examination; the last one was referred for testing but was not tested due to non-congruence of symptoms.

**Table 1:** Participants’ characteristics (N = 22)

	N		N
**Age**		**Education level**	
20–29	4	Primary	1
30–39	4	Secondary	6
40–49	3	Technical/college	6
50–59	3	University	9
60–69	4	**Civil status**	
≥ 70	4	Single	7
**Gender**		Married/common law	9
Female	13	Separated/divorced	3
Male	7	Widowed	3
Non-binary	2	**Pain Duration**	
**Province**		1–2 years	3
Quebec	7	2–5 years	4
Ontario	5	5–10 years	5
British-Columbia	4	> 10 years	10
Alberta	3	**Pain Location**	
Nova-Scotia	2	> 3 body sites	18
Saskatchewan	1	Head/face	7
**Ethnicity**		Neck	13
White	12	Shoulder	14
First Nations/Indigenous	5	Arm	5
North-African	2	Elbow	7
Black	1	Wrist	9
Asian	1	Hand	8
Metis	1	Back	17
**Living area**		Abdomen	3
Urban	15	Hip	12
Rural	7	Buttock/genitals	3
**Language**		Leg	10
English	16	Knee	11
French	6	Ankle	7
		Foot	1

### Data collection

Interviews were conducted online using Zoom Pro™, by a bilingual female research assistant trained in social sciences with additional social intervention experience in the areas of pain and mental health (VP). Participants could connect via a telephone number or a hyperlink and they were free to enable or disable their camera. Six participants chose the telephone; ten had the interview online with a camera, and six without the camera. The absence of visual contact could modify the relationship between the participant and the interviewer as non-verbal elements were less easily available. Interviews lasted between 43 and 113 min (median: 65 min). Interviews were audio-recorded and transcribed verbatim. Names, dates, places, and all information that could lead to identifying participants have been de-identified in transcripts. A few weeks after the interviews, participants were sent a CDN$50.00 compensation for their time and effort.

The interviewer used a semi-structured guide with open-ended questions and prompts covering the impact of the pandemic on participants’ chronic pain condition, pain management, psychological well-being, finances, personal and professional lives, as well as their hopes for the future. The guide was reviewed by three patient partners coming from the Quebec Association of Chronic Pain and the Canadian Arthritis Patient Alliance. Congruence between English and French versions of the guide was checked by four team members who are fluent in both languages. At the end of each interview, the interviewer produced fieldnotes describing settings and non-verbal elements. Fieldnotes included pre-analysis ideas and information on novelty/redundancy with previous interviews, to progressively assess the level of data redundancy and information power [53, 54]. After 22 interviews, the research team identified a sufficient balance between redundancy and novelty to document diverse experiences in detail and adequately address the study’s goals.

### Data analysis

Four members of the research team with diverse professional backgrounds (sociology, clinical psychology, population health) were involved in data analysis (LD, ML, MGP, AL). Our epistemological approach to data analysis in this study was based on an interpretivist perspective considering that themes are constructs derived from the analysts’ interpretation of data, and do not “emerge” directly from the interviews [54–56]. We deployed an iterative coding procedure with NVivo® Software, initially using labels based on the themes appearing in the interview guide. We progressively produced new themes and subthemes integrating the analysts’ diverse insights on interview excerpts [54, 55]. Themes were built through merging and enriching initial codes over the course of the analysis and team discussions [55] Along the analysis process, we wrote memos (analysis notes) to describe and understand variations in participants’ narratives for each code. These notes were constantly updated to compare and contrast excerpts; they helped build and deepen the themes and their dimensions [57]. All interviews were analyzed by at least two team members, and interpretations were discussed during regular team meetings. The diverse backgrounds of the analysts enriched the interpretation by providing interdisciplinary perspectives on data [52, 58]. This helped improve rigor through fostering the researchers’ reflexivity and questioning of their preconceived ideas on data interpretation. We did not seek to eliminate subjectivity in the analysis but rather to foster rigorous interpretation through the addition of several subjectivities. The quotes from francophone participants cited in this article were translated from French to English with the collaboration of a professional translator whose native language is English [59].

## Results

**Fig. 1:**
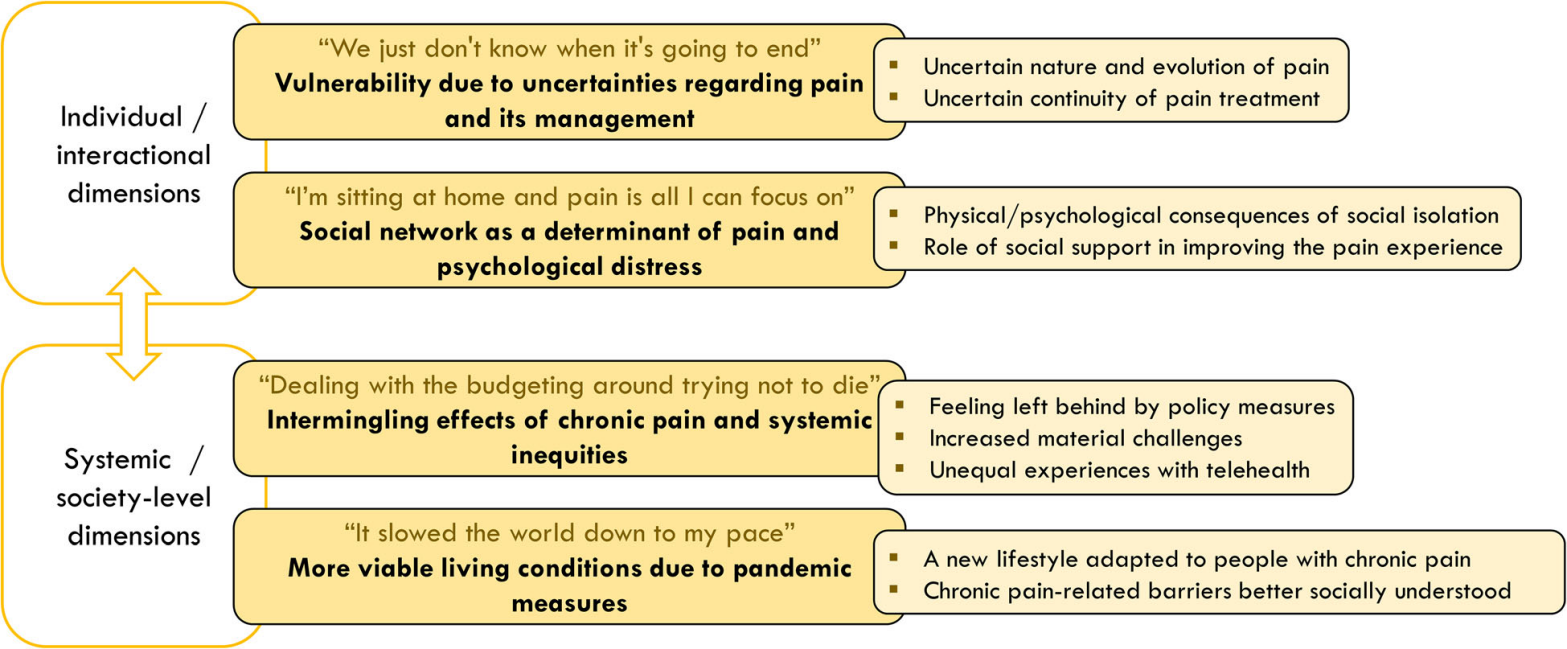
Four dimensions of the chronic pain experience during the COVID-19 pandemic

The four themes presented in this section refer to four main dimensions of the chronic pain experience during the COVID-19 pandemic. These include two individual/interactional dimensions: (a) Reinforced vulnerability due to uncertainties regarding pain and its management, (b) Social network as a determinant of pain and psychological condition during the pandemic; and two systemic/society-level dimensions: (c) Intermingling effects of chronic pain and increasing systemic inequities; (d) More viable living conditions due to pandemic measures (Fig. 1).

### “We just don’t know when it’s going to end”: reinforced vulnerability due to uncertainties regarding pain and its management

#### Uncertain nature and evolution of pain during the pandemic

Several participants were concerned with the uncertain nature and evolution of their pain in the context of the pandemic. Many of them reported increased or new pain that they linked with pandemic-related anxiety or disrupted self-management routines. For some of them, these new pain experiences could lead to uncertainty regarding a potential COVID-19 infection. They reported difficulty making the difference between flares in their usual pain symptoms and additional pain that could result from COVID-19. Since available public information described multiple and changing manifestations of the coronavirus disease, participants’ usual pain symptoms could suddenly become unfamiliar and threatening:

*I was feeling a lot more pain, and I don’t know if it’s because there was a concern “Okay, is this my regular pain or do I have COVID?”[…] It started with my throat would go hoarse and then I’ll start getting an increased cough and headaches, and I’m like “Is this the normal that I get or is it COVID?” Because so many of my symptoms are the symptoms of COVID except I don’t get a high temperature. COVID symptoms are my normal. (female, 40–49, white)*

Participants reported that they were also concerned about the evolution of their pain in the context of a broader uncertainty regarding the end of the pandemic. For example, frontline workers living with chronic pain faced intensified workload without knowing when this effort would come to an end. This situation could lead to a worsening of their chronic pain condition as their bodily resources were severely strained for a long and indefinite period:

*The pandemic affects me more because I’m a nurse in a hospital, and my working hours have increased. The workload is different. It’s had an impact [on my pain]; I have to say. It’s not just like a gastroenteritis outbreak, where we know it’ll be over in a month or two. Now we just don’t know when it’s going to end. So we keep squeezing the lemon again and again. […] It has pushed my pain up another notch, tested my pain tolerance. When I get home, I’m far more tired, my pain is much worse. (female, 20–29, White)*

#### Uncertain continuity of pain treatment

As for some other chronic health conditions, the pandemic led to new uncertainties regarding the continuity of health care services for the treatment of chronic pain. Because chronic pain conditions were not deemed as emergencies, many participants had their surgeries postponed to an undetermined date and were left with unrelieved pain. Even when healthcare resources were maintained, several participants described increased complexity of procedures to obtain services and treatment. Delays and medication shortages could unexpectedly threaten treatment continuity. This situation could add up to pre-existing barriers in obtaining a proper diagnosis and adequate treatment for pain, especially for those using opioid pain medication:

*Getting the medication is a problem. You spend more time. They only will fill out a prescription for 30 days, so every 30 days you’ve got to go back and do the whole process over again. It really honestly does not make sense. The COVID crisis should not have an adverse effect on people dealing with chronic or severe pain. […] Pain is getting worse, it’s progressively worse. But that is a whole other subject on getting me access to the medication because of the opioid crisis and all that nonsense. (male, 70–79, White)*

Uncertainty of accessing health services due to the pandemic could entail detrimental consequences for participants’ mental well-being. One participant reported he made a suicide attempt because he could no longer seek help to relieve his pain efficiently and had no perspective of improvement in the near future:

*The pain got worse, and that had an effect on my mental health, to the point where I attempted suicide a month ago. What really used to save me was my physio[therapy]. I used to go twice a week. It made me feel better. But without my physio, I’m uncomfortable all the time, not able to sleep. And when I saw my situation, I said to myself: “This is how it’s going to be until the end of my days”. […] Without COVID, I might not have attempted suicide, because without COVID I would still have physiotherapy. (male, 20–29, White)*

Several participants felt physically vulnerable to COVID-19 due to immunosuppression often associated with chronic pain conditions. Because of that, they were hesitant to use health care resources during the pandemic, which could threaten the continuity of their pain treatment. However, other participants used their experiential knowledge as chronic pain patients to feel better prepared against risks of COVID-19 exposure in healthcare services:

*I’ve been in and out of the hospital way more times than I care to remember because of surgeries, and I know that it’s not the cleanest place, hospitals do their best, but it’s not the cleanest. Yes, you can get sick in the hospital, that’s happened to me, so I speak from experience. (female, 70–79, White)*

### “Now I’m sitting at home and pain is all I can focus on”: social network as a determinant of pain and psychological condition during the pandemic

#### Numerous physical and psychological consequences of social isolation

Having a reduced social network during the pandemic was an aggravating factor for both psychological distress and physical condition among people living with chronic pain. Several participants described the vicious circle of physical pain, unavailable resources, financial problems, and social isolation that contributed to increasing their psychological challenges during the pandemic. For several of them, this situation led to suicidal ideation:

*Once I realized the financial stuff, there were just a bunch of different things that had happened shortly before the pandemic and so I ended up feeling suicidal. I ended up calling the helpline, which I’ve never done before. Usually, I would try to see a friend, or I had therapists in the past, but don’t have access. Suddenly all the different things that I was wanting to access were no longer available, and so there was a feeling of hopelessness and overwhelm. (non-binary, 30–39, white)*

In addition, several participants reported that ceasing their social activities because of the pandemic led them to focus on their pain much more than usual. That was particularly the case for participants living alone with few social contacts:

*And the worst part about it is because I was active, I like to keep active, because I have fibromyalgia, and so I’m in a lot of pain a lot of the time and if I keep active I don’t notice it. So now I’m sitting at home and [pain] is all I can focus on. My feet are killing me. I don’t have anything to distract me. (male, 50–59, White)*

Older participants seemed particularly affected by the loss of in-person interactions with family members, especially grandchildren:

*For me, the biggest thing will be getting my family together and being able to just spend time with each other without worrying social distancing, to be able to hug my kids, my grandkids. I’ve stolen a few hugs and then I’ve felt guilty because I thought, you know, “What if I put them at risk?” That’s what COVID-19 has done to us, because you don’t know. (female, 60–69, Indigenous)*

#### The important role of social support in improving the pain experience during the pandemic

 In contrast, some participants reported that the possibility to maintain social contacts during lockdown had a positive influence on their psychological state and physical pain. Some participants felt more supported during the pandemic than usual, as relatives could be more often available to offer help with their chronic pain:

*That has been a good thing because normally the person that is helping me, normally they would be at work physically. So normally if I had this call for surgery, I would have to reach out further to someone, so it would have been a lot more planning around how to get there, how to do everything. That has been a benefit that [now] I have the help right here at home. (female, 50–59, White)*

Some participants indicated that online peer-support resources, such as pain patient groups, played an important role to foster their psychological well-being during the pandemic. Several participants explained that the availability of these group was reassuring *per se*:

*My pain support group opened up the Zoom weekly things, and to be honest, I haven’t logged on, but they still once a week send me the Zoom invite for, “here’s the support group, it’s open if you want to go”. Just seeing that it’s open has been helpful because I know there’s a desire to seek community because it helps just the fear of the whole, not just having pain, but the pandemic, to communicate with people who I feel can understand. (non-binary, 20–29, white)*

### “Dealing with the budgeting around trying not to die”: Intermingling effects of chronic pain and increasing systemic inequities

#### Feeling left behind by policy measures

Several participants from social groups facing systemic inequities due to race, age, and/or disability felt forgotten by governmental measures and the healthcare system during the pandemic. Participants who were living with disability pensions due to their chronic pain condition seemed more economically precarious than those who had other sources of income. They reported that new governmental aids offered during the pandemic were often insufficient to meet their needs.

In addition, several participants felt that the risks of severe COVID-19 complications associated with their chronic pain conditions were underestimated by some healthcare providers and/or public health recommendations. One Indigenous woman reported she was mistreated by a healthcare provider who paid little attention to her chronic disease:

*One bad thing that happened with the sinus infection, I had to go for blood tests and x-rays and the lady there made me wear a mask. I didn’t mind the mask; it was just her whole attitude. […] That lady started yelling over my [family members’] heads that [my chronic disease] was not why I was wearing a mask, I was wearing a mask to protect everybody else. She was really, really rude. I reported it, but nothing ever came of it. I had a sinus infection and my doctor said that I was wearing a mask for my protection because I had lupus. That’s what my doctor told me. (female, 50–59, Indigenous)*

Another participant deplored that Indigenous communities did not benefit from the government’s new financial aid efforts despite their difficult living conditions. She highlighted the cumulated burdens of systemic racism and the COVID-19 pandemic for Indigenous people:

*In terms of community, we have a shorter lifespan, we have less resources available to us. Everybody says that Canada is such a wonderful place to live. No, not for everybody. The reality is white privilege is predominant and I don’t live in a white privileged world, I live in another world. So, has that affected my illness and how I’m feeling all the time? For sure, but that affected me long before COVID. […] I could probably go on about how COVID has affected us First Nations people generally. Even when you look at the resource distribution that the government has laid out. It’s interesting that they missed groups, Indians and seniors. (female, 60–69, Indigenous)*

#### Increased material challenges

Several participants mentioned the cumulation between their vulnerable physical condition and new material challenges faced due to the COVID-19 pandemic:

*The thing that I wanted to mention was the finances, because that has been such a prevalent thing in terms of all the aspects of the pandemic: dealing with the budgeting around trying to not die, and that has been very strange. (non-binary, 20–29, White)*

Some participants reported being in increased financial vulnerability due to job loss or already precarious situations. For some women, school closures and the need to care for children intensified economic problems. This could add to their pre-existing financial challenges due to chronic pain and disability. One participant explained that she had to temporarily stop her two jobs to care for her child during the lockdown. She faced income decrease despite governmental aids and lost her health insurance along with her job, which was challenging for her medical follow-up:

*I was doing two jobs. Now I’ve stopped both. Unfortunately, where I was full time, I had no benefits, I didn’t qualify for any of the dental, medical, but in the other [job] I did. But there, I was still on probation, so I lost it [the health insurance benefit from the second job]. And since it’s due to COVID, I don’t qualify for employment insurance, so they put me on CERB [Canada Emergency Response Benefit]. I would have been better off on employment insurance than on CERB, but (sighs), I had no choice. (female, 30–39, Black)*

In addition, housing challenges in deprived neighborhoods could negatively affect participants’ condition during the lockdown period::

*I’m not in the 70-and-over category, but I did feel a little bit vulnerable. I have a lot of health issues and I think at one point I was almost suicidal. […] Now I’m even more worried, because they say we can have 10 people over, but we have to keep our distance. Come on! [The apartments here are] 4½s, 5½s, you know. I live in [neighborhood], it’s full of immigrants. I’m one, too. Can you imagine them staying 2.5 m apart? Sure, if you have a house, a yard, but even then. (female, 60–69, North-African)*

#### Unequal experiences with telehealth services

Participants reported contrasted experiences with telehealth services. Participants who already had a stable follow-up and trusting relationship with a physician reported being overall satisfied with online consultations. They appreciated being able to talk to a doctor without the burden of going to their office and were reassured that a physician would be available in case a problem occurred. However, virtual care seemed to increase pre-existing difficulties in accessing health care and during patient/provider interactions. Participants who did not have a regular family physician or had prior unsatisfying communication with providers reported that the situation was even worse in virtual settings. Participants described increased difficulty establishing an empathetic relationship, and accessing diagnosis for pain symptoms without an in-person encounter:

*In 15 min, if I’m going to the doctor and there’s something wrong with the leg or back, or whatever, they don’t have time to look at it. Now you can’t even get in to see the doctor. It’s on the telephone, a three-minute conversation. But they’re still getting paid for the call. (male, 70–79, White)*

In addition, participants’ financial difficulties could intensify barriers to accessing online care services during the pandemic:

*With COVID, we’re supposed to do everything on the internet now, but I have never had a budget from the government to have internet or a device of any kind to access the internet. Any time that I have tried to complain about that before and say it’s an essential utility for a person with a disability, they say, “well, you can come into the office” […] So if anybody who is disabled in [my province] wants to be able to access the services that they literally require right now, they’re using debts, they’re begging, they’re going to family and friends. (non-binary, 20–29, White)*

### “It slowed the world down to my pace”: more viable living conditions due to pandemic measures

#### A new lifestyle adapted to people with chronic pain

Many participants considered that the general “slowdown” of society during the pandemic was a welcome positive aspect because this way of life was more adapted to their chronic pain condition. Several participants appreciated being able to take a break in a hectic life, as pandemic restrictions provided them with more time for self-management of pain. Some noticed an improvement in their pain condition due to relaxation and caring routines:

*I try to find time for drawing, mandalas (laughs). They do help a bit! Because they create a quiet time when you can relax. And I also think that with this type of pain, the more you’re stressed, the more you feel it. And then, when you’re doing that, you don’t think about anything else, just what you’re doing, so you feel like you have more freedom, you might say. So, yes, there’s definitely a positive side to it. (female, 30–39, Black)*

For several participants who were physically limited by chronic pain, stay-at-home orders were a form of relief as they did not need to decline social activities or to force themselves to participate in social events despite their pain:

*If I’m in pain I will just go home or just not do the activities and not explain myself as to why. I don’t think any of that has really changed much. And with the fact that nobody was seeing anybody for a while, that was nice in a way, because I didn’t have to come up with excuses anymore. It slowed the world down to my pace. That’s pretty much the upside. I wasn’t getting bombarded by people wanting to do something or hang out, so those were positives in a sense, I guess. (male, 30–39, North-African)*

#### Chronic pain-related barriers better socially understood

Several participants felt that the social and psychological consequences associated with chronic pain were more easily understood by others since the pandemic had disrupted everyone’s daily living. Other people showed more empathy towards participants’ usual challenges, as they were living similar issues themselves:

*It’s strange, the empathy that has come out of this situation where people are starting to understand the more subtle side of chronic things, like chronic pain or chronic illness, like the isolation that happens. Most people are very stressed out by it, but it’s kind of a normal for me, so it has been very interesting to see how people are suddenly realizing oh, you know, when you don’t get the opportunity to go to work or you can’t go out and have a social life, that is affecting your mental health, your physical health, everything. (Non-binary, 20–29, white)*

In addition, according to some participants, the social norms promoting productivity, which usually made them feel guilty from being physically limited by pain, were less present during the pandemic:

*I’ve been off work for a couple of years now. So for me, I was already at home, and I think in some ways it relieved a lot of stress, the stress of feeling like I need to get out and do things, because I don’t have to, because the world stopped. So I don’t have that stress of feeling like I should still be an accomplisher even though with my chronic pain and the fact that I have to find a different career from the manufacturing that I was doing. (female, 40–49, White)*

## Discussion

This pioneering qualitative study provided new knowledge about the intersecting and compounding impacts of the COVID-19 pandemic and the social determinants of health in the physical, psychological and social experience of chronic pain. In this section, we discuss our original contributions to knowledge on health inequities as well as pathways for more equitable policies and clinical practices.

### Contributions to knowledge on health inequities

This study highlighted important disparities in participants’ experiences during the pandemic, especially regarding material challenges, psychosocial impacts, social support, and barriers of access to pain management and virtual care services. These disparities need to be interpreted through the lens of social determinants of health and the wider current social, political, and economic responses to the pandemic.

First, the specific situation of people living with chronic pain regarding health care inequities during the pandemic needs to be discussed. Our study showed that for people with chronic pain, new and reinforced uncertainties regarding pain and its treatment have been adding up with preexisting health care precarity prior to the pandemic. Indeed, people living with chronic pain already faced many challenges in obtaining a diagnosis and being believed as legitimate patients by health care providers [60–64]. During the past years, many chronic pain patients have been facing increased uncertainties regarding access to treatment, as the opioid overdose epidemic entailed restrictive policies regulating pharmacological responses to chronic pain [65–67]. In this context, chronic pain itself could create discrimination and barriers to accessing healthcare services [68]. A recent review suggested that the COVID-19 pandemic and the opioid overdose epidemic had compounding detrimental effects on the treatment of chronic pain [69]. Our study underscored that chronic pain and the COVID-19 pandemic could have intermingling impacts on physical, psychological, and social experiences.

Furthermore, socio-economic situation, race, and gender intersected with the political responses to the pandemic to influence the situation of people with chronic pain. As testified in the literature, chronic pain can lead to socio-economic difficulties, and people with lower socio-economic status and/or facing marginalization are at increased risks for developing chronic pain and experiencing barriers to accessing pain management [23, 26, 27, 38, 70, 71]. The present study suggested that the COVID-19 pandemic tended to aggravate this already challenging situation, as socio-economic inequities could negatively influence physical and mental health experiences for people with chronic pain during the pandemic. Participants with few financial resources experienced numerous additional challenges including intensified anxiety and difficulty accessing virtual care services. Poor housing conditions in socio-economically deprived neighborhoods were mentioned as additional sources of stress regarding risks of infection with COVID-19. Those in precarious working situations were especially at-risk for losing health insurance during the pandemic, which could negatively affect their health care trajectory for chronic pain and other health conditions.

In addition, previous research showed that persons from racial minorities and Indigenous Peoples face specific economic challenges and barriers to accessing healthcare [72–74]. In the area of chronic pain, several studies have highlighted racial inequities regarding pain assessment and access to analgesia, with racialized persons facing prejudices, stigma, and lack of recognizing of their pain [27, 32, 75]. According to a recent study, Black Americans with chronic pain were particularly at risk of experiencing higher pain levels and disruptions in mood and sleep during the COVID-19 first wave [45]. By highlighting the specific challenges of belonging to a racial minority with chronic pain during the pandemic, our results contribute to situate their deteriorated pain outcomes within social experiences and systemic barriers. Indeed, in our study, racialized and Indigenous participants experienced discrimination in health care and felt forgotten by the government’s financial aid measures. Several of them reported being more financially precarious due to the pandemic context.

Regarding gender inequities, some women in our sample, especially those with lower socio-economic status and/or belonging to racial minorities, seemed to face additional challenges related to gender expectations and roles. Some of our female participants reported facing new family care responsibilities in addition to their chronic pain. Such intersectional inequalities during the pandemic have been noticed in the general population [76]. In addition, women are more present in frontline health care work; they also often endorse care and disease prevention tasks in their families. Regarding the two non-binary participants in this study, they cumulated several increasing health and social challenges such as disabilities and financial precarity. In the previous quantitative investigations of pandemic impacts on chronic pain, the situation of non-binary people could not be documented due to insufficient data [43–46]. According to results from the COVID-19 and Chronic pain Pan-Canadian quantitative study, changes in pain treatment due to the pandemic were more frequent among women than men [46]. This suggests that women experienced specific difficulties in accessing their usual pain treatment. In an American study, no differences were found between women and men in terms of changes in pain severity and interference in daily life during the COVID-19 first wave [45]. However, the authors state that differential impacts will likely appear over time, given the existing literature documenting gendered disparities related to pain [45]. Indeed, inequities have been documented in the experience and treatment of chronic pain: women are more represented in underdiagnosed or medically controversial conditions such as fibromyalgia and they are more exposed to providers’ prejudices during health care interactions [29, 64]. For example, providers typically consider that women with chronic pain, especially women of color, have exaggerated complaints, and physicians are more likely to attribute psychological causes to women’s physical pain [29, 64, 77]. Suffering from chronic pain therefore adds another source of vulnerability to gendered and intersectional inequities [77]. By documenting barriers among women and non-binary people with chronic pain during the pandemic, our study can support the development of further longitudinal work on pandemic impacts on gendered inequities and pain.

An important finding in our study is the suspension, during the lockdown period, of many social expectations that people in pain usually dealt with. Several participants felt that during the pandemic, the world was more aligned with their physical capabilities, and other people were more empathetic with their situation. This finding echoes with sociological and anthropological studies of the chronic pain experience highlighting how much this health condition requires reorganizing one’s life around pain, and grieving prior activities and interests [78–81]. Inability to meet social expectations, such as socializing or productivity, can be a source of stigma for people with chronic pain [77, 82, 83]. People with chronic pain regularly experience dilemmas when dealing with the presence of pain during social interactions, as they need to choose between being physically relieved, or enduring painful situations to preserve their social relationships [84]. Paradoxically, the positive outcomes reported by several participants during the pandemic tend to reveal how much the pre-pandemic “normal” was challenging for people living with chronic pain. This suggests that there is still a long way to go for the full inclusiveness of people with chronic pain in society, in terms of concrete adaptations of working/leisure activities, but also in terms of value change regarding collective representations of people in pain’s contribution to their community. As shown by existing research in critical disability studies, it is essential to consider the experience of disability as a social and political issue instead of a problem located in an individual’s body [85]. Indeed, in neoliberal societies, disability and vulnerability are often viewed as individual deficits, which leads to increased social exclusion [86, 87]. Our findings suggest that the pandemic-related “slowdown” led to a form of temporary re-balancing of power in society by removing some of the usual norms and requirements on people living with chronic pain. Indeed, the place that societies give to people living with disabilities – including the limitations caused by chronic pain– is a major issue for social equity. This intersects with other forms of systemic inequities such as those based on race, gender, and socio-economic status [88].

### Implications for policy and practice

This study had important implications for health and welfare policies, as well as clinical practices. This study provided policymakers with concrete examples of lived experiences showing the multiple ways in which chronic pain intersects with social inequities during the COVID-19 pandemic. This is essential to catalyze the development of future policies with a more diverse input. Healthcare and social welfare policies need to address the issues of systemic inequities and social inclusiveness, in order to improve the situation of people living with chronic pain during and beyond a pandemic such as COVID-19.

Shaping welfare policies that would acknowledge the beneficial outcomes of a general “slowdown” in social productivity injunctions could positively affect people living with chronic pain as well as the general population. To improve the social inclusiveness of people with chronic pain, priority should be given to ensuring them sufficient quality of life. It is essential to improve the recognizing of the experience of chronic pain and related physical limitations, through information and advocacy to the public, health care providers, and policymakers, as well as material compensation of pain-related disabilities. This could be beneficial to people living with chronic pain in terms of material living conditions and, more broadly, social acceptability of their physical limitations.

In addition, reducing social inequities towards pain management requires policies addressing socio-economic gaps in access to health care services, especially those that are not covered by public healthcare insurances (e.g. non-pharmacological resources). Racial and gendered bias in pain management, which have been shown to be common among providers [29, 75], also need to be addressed through the promotion of equity-oriented approaches to pain management training and practice. Some recent initiatives promoting equity-oriented health care [28, 89] could be promising pathways providing concrete guidance for providers to include systemic dimensions of health care in their practice, through trauma-informed care, cultural safety, and harm reduction [28]. Implementation of such approaches in clinical settings needs to be supported by proactive public health policies establishing health care equity as a priority.

It is also urgent that policymakers take action to prevent the pandemic’s detrimental effects on the mental health of people living with chronic pain, especially the most socially and economically vulnerable. Implementing accessible psychosocial interventions tailored to this vulnerable population appears essential. Our findings suggested that virtual peer-support resources may be interesting pathways to prioritize. However, to achieve such a goal, policymakers will need to take measures to provide universal and affordable access to the internet and technological devices. Otherwise, the gap in health equity will most likely be furthering [90] with damaging consequences for the lives of people living with chronic conditions and lower socio-economic status, functional limitations, or belonging to a minority group. Welfare and healthcare policies need to provide support –including financial and technical resources – to help disadvantaged populations accessing proper virtual care services.

### Study strengths and limitations

This study covered a large territory including urban and rural areas of the most populated geographic regions of Canada. This enabled us to analyze the impacts of the pandemic on people with chronic pain with diverse socio-demographic characteristics and living in various Canadian settings. However, we could not recruit participants from areas that were less represented in our quantitative sample, such as the Territories. The specific situation and challenges of people with chronic pain in these areas remain unknown. Our findings can be used to design future studies in these populations.

Furthermore, this study provided important findings regarding the intersection of chronic pain with systemic inequities during the pandemic. However, one limitation of our approach to inequities is that it may sometimes be difficult to demonstrate with certainty the link between social determinants and participants’ individual situation, especially when participants do not explicitly refer to their social, racial or gendered condition during interviews. However, as the analysis of systemic inequities in health care proved to be a valid and relevant interpretation framework in the literature, we believe that it is crucial to adopt such lens when interpreting our data, to highlight the structural factors influencing the chronic pain experience during the pandemic. Future mixed-methods studies among people living with chronic pain should prioritize sample representation of minorities in order to provide population-based data in this domain along with in-depth analyses of participants’ experiences.

## Conclusions

This qualitative study revealed the multiple impacts of the COVID-19 pandemic in the daily living of people with chronic pain as well as the major influence of several types of intersecting systemic inequities on their situation. The pandemic has intensified many preexisting disparities and challenges faced by people with chronic pain at social, physical, and psychological levels. It is more than ever urgent to rethink health and welfare policies, as well as services and interventions, in order to address socio-economic, racial, gendered, and disability-related inequities and offer more inclusive and equitable living conditions to people with chronic pain facing systemic barriers.

## Data Availability

The data that support the findings of this qualitative study are not publicly available due to ethics requirements.
